# Alterations of the Gut Microbiome and TMAO Levels in Patients with Ulcerative Colitis

**DOI:** 10.3390/jcm13195794

**Published:** 2024-09-28

**Authors:** Yelena Laryushina, Nadezhda Samoilova-Bedych, Lyudmila Turgunova, Samat Kozhakhmetov, Assel Alina, Maxat Suieubayev, Nurislam Mukhanbetzhanov

**Affiliations:** 1 Department of Internal Diseases, Karaganda Medical University, Karaganda 100000, Kazakhstan; laryushina@qmu.kz (Y.L.); turgunova@qmu.kz (L.T.); alina@qmu.kz (A.A.); 2 National Laboratory Astana, Nazarbayev University, Astana 010000, Kazakhstan; skozhakhmetov@nu.edu.kz (S.K.); maxat.suieubayev@nu.edu.kz (M.S.); nurislam.mukhanbetzhanov@nu.edu.kz (N.M.)

**Keywords:** ulcerative colitis, UC, inflammatory bowel disease, IBD, thrymetilamin-N-oxide, TMAO, gut, gut microbiome, microbiota sequencing, gut microbiota metagenomic sequencing

## Abstract

**Background:** Ulcerative colitis (UC) is an idiopathic and heterogeneous large intestine disease, characterized by chronic mucosa and submucosa inflammation. Alteration of the intestinal microbiome in UC may be responsible for modifications in metabolite production. Aim: To investigate the microbiota status and trimethylamine-N-oxide (TMAO) metabolite levels in patients with UC according to clinical and endoscopic activity. **Methods:** As part of a grant project AP14871959 from September 2022 to October 2023, 31 patients with UC and 15 healthy volunteers over 18 years at the Clinic of NCJSC “KMU” were assessed for blood TMAO level and metagenomic sequencing of fecal microbiome. **Results:** A significant depletion of the main representatives of Bacteroides, Parabacteroides, Prevotella; and an increase in the relative abundance of the genera Actinomyces, Klebsiella, Limosilactobacillus, Streptococcus, Escherichia-Shigella were detected in patients with UC. The number of p_Actinobacteria (g_Collinsella) and p_Eubacterium (g_Xylanophilum) representatives with genes encoding TMA-trimethylamine conversion is significantly reduced in UC patients. TMAO levels were significantly lower in UC patients than in healthy individuals (0.233 µmol/L, *p* = 0.004). TMAO decreased with disease severity and significantly differed between patients with different activities (*p* = 0.034). **Conclusions:** The composition of the intestinal microbiome changes and the level of TMAO decreases in patients with UC at different activities.

## 1. Introduction

Ulcerative colitis (UC) is a chronic inflammatory bowel disease characterized by long-term inflammation and ulceration of the colon’s lining. Recently, there has been increased interest in the role of the gut microbiome in the development of UC. Studies have shown that an imbalance in the composition of gut bacteria can be both the cause and consequence of inflammation in the intestines [1].

Many studies have aimed to understand the composition of the gut microbiota in people with UC, and they have found significant changes in the numbers of bacteria from three main phylum: Bacteroidetes, Firmicutes, and Proteobacteria [2,3,4]. In a recently published review, general trends were found in the decrease in beneficial bacteria with anti-inflammatory properties. Patients with IBD have decreased *Faecalibacterium prausnitzii*, *Roseburia*, *Bacteroides*, *Ruminococcaceae* and *Lachnospiraceae*, while harmful bacteria such as *Escherichia coli*, *Clostridioides difficile*, *Enterobacteriaceae*, and *Proteobacteri* increased [5]. Changes in the intestinal microbiome are influenced by many factors, including the administration of different therapies, the severity of the disease, and the age of the patient [5,6]. These changes in gut flora are associated with a decrease in the protective mucus layer in the intestine, which can lead to worsening symptoms in patients [7,8]. Dysbiosis caused by shifting ratios of harmful to beneficial bacteria exacerbates inflammation and correlates with increased disease activity [5].

The intestinal flora also influences the risk of developing many different diseases by producing metabolites. Researchers have focused on trimethylamine-N-oxide (TMAO), the final product of trimethylamine (TMA) oxidation, metabolized by the intestinal microbiota. Previous studies have shown its role in the pathogenesis of cardiovascular, autoimmune, metabolic, and other diseases [9,10,11,12,13,14]. Studies are ongoing to understand changes in TMAO levels in ulcerative colitis (UC). Wilson et al. found a decrease in TMAO levels in patients with UC compared to healthy individuals, and considered TMAO a potential biomarker for IBD [15]. Using only clinical assessment of disease activity, they found that TMAO was lower in active disease than it was in inactive colitis. Subsequently, in 2023, Kul Seref et al. assessed the relationship between TMAO, inflammatory markers, and endothelial and coronary microvascular dysfunction. They found a decrease in TMAO in patients with inactive inflammatory bowel disease (IBD), but these findings were not statistically significant [16]. There are currently no studies examining the changes in the intestinal microbiome composition and the level of the TMAO metabolite in patients with ulcerative colitis (UC).

## 2. Materials and Methods

### 2.1. Study Design

This was an observational, cross-sectional study conducted at the clinic of the NCJSC “Karaganda Medical University”. Participants were recruited from September 2022 to October 2023 and 46 individuals over the age of 18 with no mental or severe neurological conditions, of both genders and without restrictions based on race or ethnicity were enrolled. All participants signed an informed consent form confirming their willingness to participate in the study, and were screened for eligibility. Additional criteria for inclusion in the main group included the absence of acute conditions or exacerbations of chronic diseases, as well as the avoidance of taking antibacterial medications for 6 months before the study, and non-steroidal anti-inflammatory drugs (NSAIDs) and probiotics for 3 months prior. In the main group, participants were required to have a confirmed diagnosis of ulcerative colitis based on a combination of clinical, laboratory, endoscopic, and histological findings. Naive patients whose diagnosis was uncertain were not included in the study. The selected patients were all residents living in central Kazakhstan, specifically in the Karaganda region.

The protocol for the study was approved by the Local Ethical Committee of the NCJSC “KMU” (Meeting Minutes No. 1 dated 20 September 2022). A control checklist of items in report is included in Supplementary Materials.

During the clinical trial, each participant was surveyed using the GIVES-21 nutrition screener, to assess their diet over the previous week. The questionnaire included 9 questions about the types of food they ate in each category and how much they consumed [17].

### 2.2. Sample Collection

Each participant in the study collected two fecal samples in sterile containers at home and then visited a gastroenterologist within one hour of defecating. One container of feces was immediately frozen at −80 °C after collection from the patient. The initial frozen fecal samples were then transported to the Life Sciences Center at the National Laboratory of Astana, Nazarbayev University. All participants underwent a clinical examination that included an assessment of their current condition, anthropometric measurements, an interview, and a review of their medical history by gastroenterologist visit. The examination also involves filling out an MS Excel (Version 2306 (Build 16529.20154)) database.

After the examination, patients underwent blood sampling in a procedure room. An experienced nurse collected blood in three tubes: two tubes containing anticoagulant (EDTA) for general blood analysis and TMAO, and one tube with a coagulation activator for biochemical blood analysis. Two tubes of blood and one container of feces were then delivered to the Clinical-diagnostic laboratory (CDL) ”Olymp” for testing.

### 2.3. TMAO Level Determination

Peripheral blood parameters were determined on high-performance automatic 6-diff hematological analyzers of the Sysmex XN-2000 (Sysmex Corporation, Hyogo Kobe, Japan) and Beckman Coulter (Beckman Coulter, Brea, CA, USA) brands. The ELISA method was used to measure fecal calprotectin concentrations using the EliA Calprotectin 2 test system from Phadia GmBH (Thermo Fisher Scientific, Freiburg, Germany).

Blood sampling to determine the level of TMAO was performed by venous blood collection in K2EDTA vacutainers after a 12 h fast. The whole blood was immediately centrifuged (3000 rpm for 15 min), and the resulting plasma was frozen and stored at −80 °C in ultra-low temperature freezers, until analysis.

The study utilized trimethylamine N-oxide (95%, Sigma-Aldrich, St. Louis, MO, USA), formic acid (95%, Sigma-Aldrich, St. Louis, MO, USA), acetonitrile (99.9%, Sigma-Aldrich, St. Louis, MO, USA), and water with a high purity level (18.2 mg/L) obtained from the Milli-Q (Millipore) system. For analysis, 100 microliters of plasma were used. To this, 600 microliters of acetonitrile were added, followed by centrifuging at 20,000× *g* for 10 min at 4 °C. The resulting supernatant (100 microliters) was transferred to a vial, and 100 microliters of water were added. The solution was then injected into an HPLC-MS/MS system, with a sample volume of 10 microliters. The analysis was performed using an Agilent 1260 Infinity HPLC system (Agilent Technologies, Santa Clara, CA, USA) and a G6130A Quadrupole LC/MS (Agilent Technologies, Santa Clara, CA, USA). A ZORBAX Eclipse XDB (Agilent Technologies, Santa Clara, CA, USA) 80 °C 18 column, 2.1 × 75 mm, with a particle size of 3.5 microns, was used for separation. A Zorbax Eclipse XDB C-18 protective column, 12.5 × 4.6 mm, with the same particle size, was also used. The separation was carried out isocratically using eluents A and B, which were 0.125% formic acid solutions in acetonitrile and water, respectively, in a 1:1 ratio; at a flow rate of 0.250 mL/min and a column temperature of 30 °C [18].

### 2.4. Endoscopic Examination

Patients with ulcerative colitis were scheduled for a colonoscopy within 7 to 10 days after their initial visit. The procedure was performed by an experienced endoscopist who had more than 5 years of expertise with IBD patients. The examination was performed using an Olympus CF-H170L colon videoscope (SN 282303- Olympus, Shinjuku, Tokyo, Japan). The outcome of the procedure was assessed using the Mayo Endoscopic Score (MES), which ranges from 0 to 3. A score of 0 indicates inactive disease with normal mucosal appearance, while scores 1, 2, and 3 correspond to mild, moderate, and severe disease, respectively [19]. After endoscopic examination, a comprehensive assessment of disease activity was performed using the Mayo Index, which considers clinical and endoscopic data. All patients were divided into two groups based on their total score: Group 1 (UC-1) consisted of patients with a total score between 0 and 5, while Group 2 (UC-2) included patients with a Mayo index score of 6 or higher.

### 2.5. Microbiome Analysis

After delivery to the laboratory, fecal samples from ulcerative colitis patients and control volunteers were placed in DNA/RNA Shield-Fecal Collection Tube kits (Cat. No: R1101, Zymo Research Corporation, Tustin, CA, USA) with a total volume of 10 mL, according to the manufacturer’s instructions. For microbial DNA purification, ZymoBiomics DNA Microprep (Cat. No: D4300, Zymo Research Corporation, Tustin, CA, USA) was used. Strictly 0.2 mL of sample was extracted for standardized DNA extraction. Total DNA extraction was checked in 1% agarose gel, and DNA concentration was measured using a Nabi UV/Vis Nano Spectrophotometer (MicroDigital, Gyeonggi-do, Seongnam-si, Republic of Korea). 16S Amplicon Metagenomic Sequencing was performed in-house on the Illumina NovaSeq6000 platform according to and metagenomic sequencing protocol. Primary bioinformatic analysis was performed using less operational taxonomic unit scripts 2 (LotuS2).

### 2.6. Statistical Analysis and Visualization of the Results

Data entry and primary statistical processing were performed using the MS Excel program from the Microsoft Office (Version 2306 (Build 16529.20154)) software suite. Further, the patient’s data were analyzed in IBM SPSS Statistics 22. The Kolmogorov–Smirnov (K-S test) test was used to assess the normal distribution of the data. Data with normal distribution were described by Mean with standard deviation, if data are not normally distributed by Median and interquartile range. The data were compared using parametric and nonparametric criteria based on the normality of distribution. The chi-square test was used to analyze qualitative data, and the Mann–Whitney U test and Kruskal–Wallis test were used to analyze quantitative data. The Spearman correlation analysis test was utilized to calculate the mutual relationships. A *p*-value less than 0.05 was considered statistically significant (significance level α = 0.05).

The distribution of key study population characteristics by groups (CN—control, UC-1—early-stage ulcerative colitis patients, UC-2—late-stage ulcerative colitis patients) was analyzed using the Kruskal–Wallis test from the Scipy Library version 1.13.0 and maaslin2. Threshold values for q-value and *p*-value were set at <0.05. For post-hoc analysis, Dunn’s test (scikit-posthocs version 0.9.0) was used.

Data normalization was performed by filtering taxa based on the threshold value of relative abundance and filtering taxa based on the threshold variability of relative abundance.

Alpha diversity was assessed using the Shannon, Simpson, and Observed indices with the scikit-bio package version 0.6.0. Statistical data were calculated using the statsmodels library version 0.14.1.

Beta diversity was assessed using the Bray–Curtis and Canberra metrics, and data were transformed using the Hellinger transformation. Ordination was visualized using principal coordinate analysis (PCoA). For analysis and visualization, the scikit-bio library version 0.6.0 and matplotlib version 3.8.4 were used. PERMANOVA and ANOSIM results showed differences between groups.

LEfSe version 1.1.2 was used to identify the most differentially distributed taxa. LEfSe (Linear Discriminant Analysis Effect Size) allows for identifying taxa that differ between groups based on their relative abundance.

Visualization was performed using seaborn version 0.13.2 and matplotlib version 3.8.4. Additionally, logarithmic normalization was used to construct heatmaps.

## 3. Results

The present study included 31 patients with a confirmed diagnosis of ulcerative colitis and 15 controls without UC. Patients’ characteristics are presented in Table 1. Patients in the main and control groups did not differ significantly in terms of gender, age, or residence. Nutritional assessment revealed that patients with ulcerative colitis (UC) were less likely to consume alcohol, legumes, fresh fruits, and vegetables compared to the control group. The diets of the respondents did not differ in terms of the consumption of choline-, carnitine-, and phosphatidylcholine-containing foods. Both groups consumed similar amounts of cereals (including bread, cereals, and pasta), meat, fatty fish, seafood, dairy products, and eggs.

In terms of laboratory parameters, UC patients had lower albumin levels and higher C-reactive protein (CRP) and fecal calprotectin levels compared to healthy individuals. A large proportion of UC patients were diagnosed with pancolitis. According to the Mayo Index of Clinical and Endoscopic Activity (remission (0–1)—2 patients, mild disease (2–5)—6 patients, moderate disease (6–10)—18 patients, severe disease (>10)—5 patients), all patients were divided into two groups based on the sum of their points. The Mayo ≤5 points group (UC1) consisted of 8 patients and the Mayo >6 points group (UC2) consisted of 23 patients.

### 3.1. Evaluation of the Intestinal Microbiota in Patients with Ulcerative Colitis and Its Changes in Different Clinical and Endoscopic Stages

A total of 8,594,797 taxonomic marker tags were analyzed, with 7,277,914 sequences remaining after sequence denoising, trimming, and chimera filtration. The average sequencing depth was 179,273 reads per sample (interquartile range [IQR] 171,809–190,796). Using distance similarity ≥97%, the sequences were grouped into 2306 ASVs.

**Figure 1:**
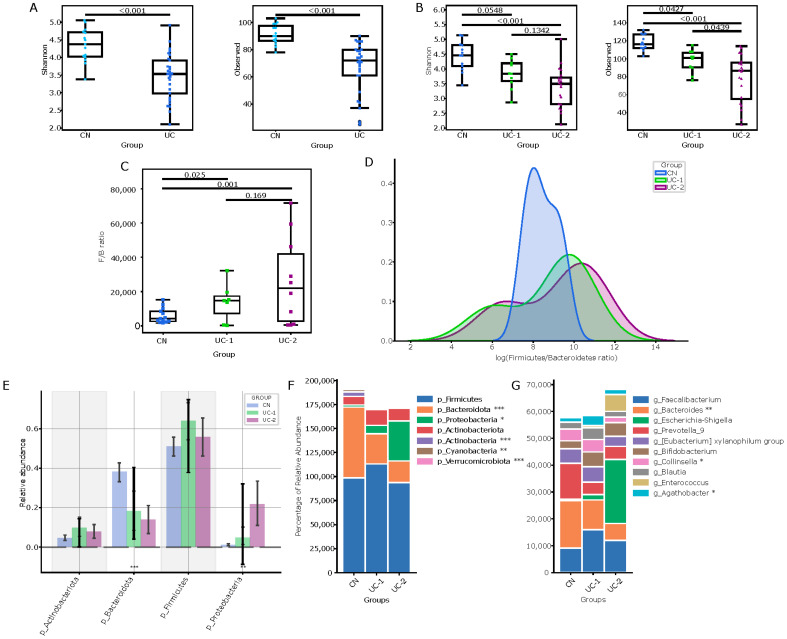
Comparative analysis of intestinal microbiome in patients with ulcerative colitis and control group. (**A**) Visualization of microbiome analysis results comparing patients with ulcerative colitis (UC) to the control group (CN). (**B**) Comparison of intestinal microbiome diversity indices in patients with varying severity of ulcerative colitis activity (UC-1 and UC-2) versus the control group. (**C**,**D**) Firmicutes/Bacteroidetes ratio in the CN, UC-1, and UC-2 groups. (**E**) Relative abundance of major bacterial phylum across comparison groups. (**F**) Stacked histogram depicting taxonomic composition at the phylum level across all groups. (**G**) Genus-level taxonomic composition comparison between the control group (CN) and ulcerative colitis groups (UC-1, UC-2). Statistically significant differences between groups are indicated by asterisks (* *p* < 0.05, ** *p* < 0.01, *** *p* < 0.001).

To assess the gut microbiota and understand its community structure and taxonomic diversity in patients with ulcerative colitis (UC), we sequenced the V3/V4 region of the 16S rRNA gene and annotated the resulting amplicon sequences using the LotuS2 pipeline. We then compared the taxonomic diversity between patients with UC and healthy controls (CN) by analyzing the Shannon uniformity and richness indices at the genus level (Figure 1A). The results showed significant differences between the two groups (*p* < 0.001 for both indices). Additionally, the observed richness index was also significantly different (*p* < 0.001).

Permutational multivariate analysis of variance (PERMANOVA) with 999 permutations was used to assess differences in the composition of the microbiota between the two groups, which showed significant differences (*p* < 0.001) between them. Comparison of the alpha diversity of the groups grouped by the Mayo activity index (0–5 (UC1) and >6 (UC2)) also showed significant changes in the microbiota, depending on disease activity (Figure 1B). In the group with more severe activity, diversity was lower (UC1/UC2, *p* = 0.0439). The higher the score, the lower the diversity. The Shannon index mean difference (case/control −0.8834), 95% CI (−1.4214 to −0.3455), *p* = 0.0006), and the Observed index (mean difference, case/control −23.7126), 95% CI (−37.5579 to −9.8674), *p* = 0.0003) showed significant differences.

Analysis of patients’ fecal microbiota between both ulcerative colitis (UC) and control (CN) groups, and comparison of mean diversity in groups of patients with different degrees of ulcerative colitis activity (Mayo Index) and controls revealed significant changes in bacterial taxa, depending on disease activity. The Observed index showed a significant decrease in diversity between the CN and UC-1 groups (*p* = 0.0427), and between the CN group and UC-2 group (*p* < 0.001), with a more pronounced decrease in the UC-2 patients compared to the UC-1 group (UC-1/UC-2, *p* = 0.0439). The Shannon index also showed differences between groups: CN/UC-1 (*p* = 0.0548) and CN/UC-2 (*p* < 0.001) (see Figure 1B). These differences were also evident in the analysis of the proportional abundance of two dominant types of bacteria, Firmicutes and Bacteroidetes, in the control group compared to groups with active ulcerative colitis. The F/B ratio was significantly different between these groups (CN/UC-1, *p* = 0.0025; CN/UC-2, *p* < 0.001) (Figure 1C,D).

There was a decrease in the relative abundance of certain bacterial taxa at the level of the phylum Bacteroidota (*p* < 0.005) and the genus Bacteroides (*p* < 0,01), which tended to decrease from remission to active disease. (CN—0.091, UC-1—0.064, UC-2—0.041). At the same time, Proteobacteria showed the opposite dynamics associated with an increase in the relative abundance of the genus Escherichia-Shigella (CN—0.002, UC-1—0.011, UC-2—0.123) (Figure 1E–G).

**Figure 2:**
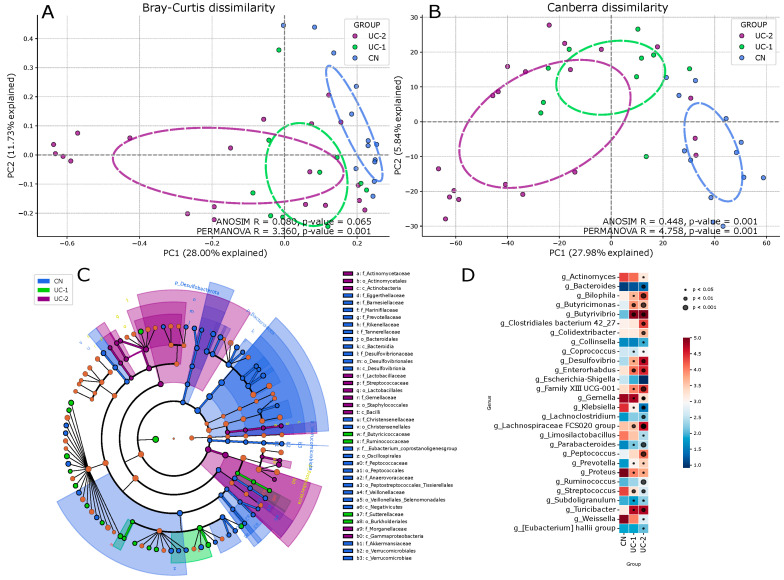
Analysis of microbiota beta diversity and differential taxa abundance in patients with ulcerative colitis (UC) and controls (CN). (**A**) Visualization of beta diversity using principal coordinates analysis (PCoA) based on Bray–Curtis and (**B**) Canberra metrics; (**C**) Cladogram showing differential abundance of bacterial taxa at genus level between groups; (**D**) Log-Scale Heatmap of Mean Abundance.

Interindividual dissimilarity in microbiota composition (beta diversity) was assessed between each pair of groups using permutational multivariate analysis of variance (PERMANOVA, 999 permutations). Partial overlap between groups was observed using the Bray–Curtis metric, but statistical tests revealed distinct differences in Figure 2A (ANOSIM R = 0.080, *p* = 0.065; PERMANOVA R = 3.360, *p* = 0.001). Clustering in principal coordinates analysis (PCoA) using the Canberra dissimilarity metric more clearly visualized statistically significant differences in Figure 2B (ANOSIM R = 0.448, *p* = 0.001; PERMANOVA R = 4.758, *p* = 0.001).

**Figure 3:**
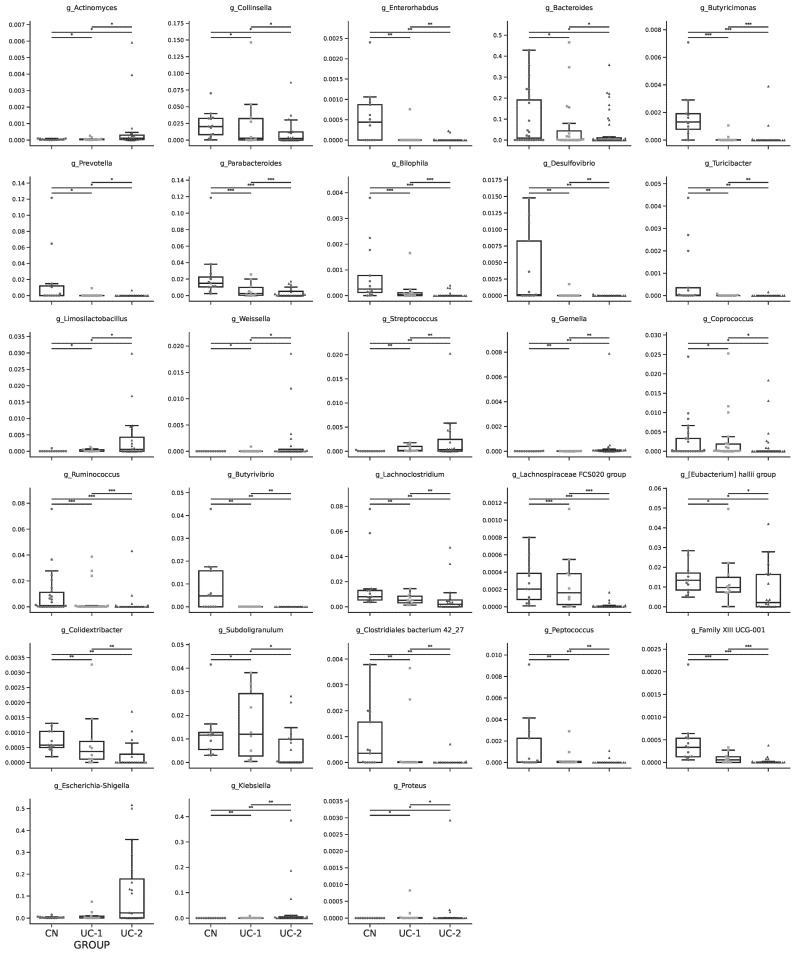
Analysis of maaslin2 beta diversity in the control group and patients with UC. * *p* < 0.05, ** *p* < 0.01, *** *p* < 0.001.

Calculation of differential microbiota abundance at the genus level presented in the cladogram (Figure 2C) and relative taxa abundance (Figure 2D) plotted against maaslin2 results revealed several significant differences according to disease activity in genus richness (Figure 3). It can be observed that the CN control group was dominated by the genera Bacteroides, Parabacteroides, *Prevotella* from the family *Bacteroidaceae*; whereas the UC-1 and UC-2 groups showed a depletion of these genera and an increase in the relative abundance of the genera *Actinomyces*, *Klebsiella*, *Limosilactobacillus*, *Streptococcus*, *Escherichia-Shigella*, depending on disease activity. In addition, there was a significant enrichment of Escherichia-Shigella in the moderate to severe group, whereas in contrast *Enterorhabdus*, *Butyricimonas*, *Ruminococcus*, *Butyrivibrio*, *Lachnospiraceae* FCS020 group, *Clostridiales bacterium* 42_27 was significantly depleted.

Thus, beta diversity analysis combined with maaslin2 analysis showed that the composition of the microbiota in patients with ulcerative colitis differs significantly from the control group, with differences observed not only between healthy and diseased patients but also between patients with different degrees of disease activity.

### 3.2. Evaluation of TMAO Levels in Patients with Different Clinical and Endoscopic Activities

**Figure 4:**
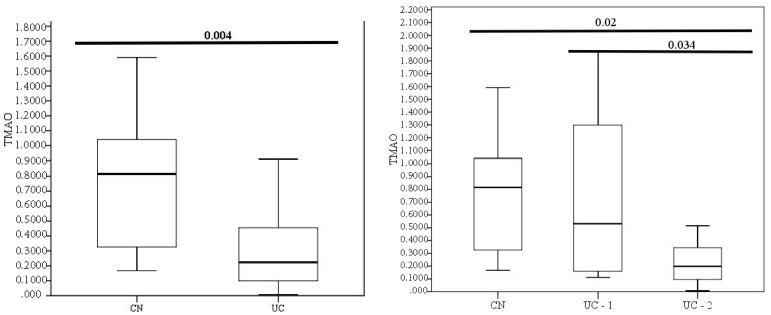
The level of TMAO in the control group and in patients with different clinical and endoscopic activity of UC.

Comparing medians in groups of healthy individuals and patients with ulcerative colitis showed significant differences in TMAO content. Moreover, TMAO level in healthy individuals was significantly higher Me 0.815 µmol/L (0.315;1.076) than in patients with UC Me 0.233 µmol/L (0.098;0.458) and had significant differences (*p* = 0.004) (Figure 4).

Dividing the patients into groups (by Mayo’s clinical and endoscopic index) also demonstrated a difference in TMAO levels in the groups with less and more severe activity. In group 1, which included patients with remission (0–1 points) and minimal activity (2–5 points), the median TMAO was 0.531 µmol/L (0.1528;1.491). In group 2 with moderate to severe activities, the TMAO value was Me 0.198 µmol/L (0.0946;0.383). The groups differed significantly in TMAO level (*p* = 0.034), as well as from healthy patients (*p* = 0.02).

The correlation of TMAO with clinical, laboratory, and endoscopic data is presented in Table 2. Laboratory parameters such as albumin r = 0.451, *p* = 0.011, and fecal calprotectin r = 0.458, *p* = 0.001 showed the most significant relationship with TMAO.

In patients with UC, there was a significant inverse relationship between TMAO and clinical and endoscopic activity (r = 0.387, *p* = 0.031). In patients with more severe activity, TMAO decreased. At the same time, TMAO did not correlate with lesion extent and such peripheral blood parameters as hemoglobin, erythrocyte count, and CRP.

## 4. Discussion

The progressive incidence of inflammatory bowel disease (IBD) and UC [20,21,22,23] is a global world problem that requires a search for new approaches to diagnosis and treatment. The development of metagenomic sequencing has allowed the accumulation of data on microbial communities in Western countries, but industrialization and globalization are contributing to the spread of IBD in Eastern and Asian countries [17,24,25]. Many studies demonstrate differences in the diversity and abundance of intestinal microflora between patients with UC and healthy individuals [26,27]. Common trends have been observed in the form of an increase in Proteobacteria and a decrease in Firmicutes [28] and Bacteroidetes-type bacteria [29]. These changes may contribute to worsening symptoms and disease progression [30]. A systematic review by Alexandrescu L. et al. showed the main patterns of changes in the intestinal microbiome in patients with IHD and emphasized the role of “healthy microbiota” in the course of the disease under different therapeutic options. The authors concluded that the correction of intestinal microbiota composition contributes to the improvement of treatment efficacy and further prognosis of patients [5].

In our study, we characterized for the first time the composition of the microbiome and the level of TMAO in patients with ulcerative colitis (UC) of different clinical and endoscopic activity, as well as healthy individuals living in the central region of Kazakhstan. Our assessment of taxonomic diversity revealed reliable differences in the uniformity and richness of the microbiota at the genus level. We found a decrease in representatives of the Bacteroides genus and an increase in the number of Proteobacteria, which is consistent with the results of previous studies. Additionally, our results showed significant differences in the composition of the intestinal microbiome between groups of patients in remission and with low disease activity, and those with moderate and severe disease activity. Groups with different levels of clinical and endoscopic activity exhibited distinct characteristics compared to healthy individuals.

As in previous studies, we found significant differences in alpha diversity between healthy and UC patients [27]. Our data also showed that impoverishment of the microbiome composition increases with decreasing disease activity, demonstrating the depletion of the defense mechanism of the colonic mucosa and reflecting the degree of inflammation [7]. We also found changes at the phylum and genus level between healthy individuals and UC patients, as in the study by Alam et al. [31]. While most studies show a predominance of representatives of the genus Bacteroides, and an increase in representatives of the genus Proteobacteria [27,28,29], in the case of our patients we found a decrease in the number of representatives of the genera Bacteroides, Parabacteroides, and *Prevotella* of the family *Bacteroidaceae*; with a simultaneous increase in the number of the genera Actinomyces, Klebsiella, *Limosilactobacillus*, Streptococcus, and Escherichia-Shigella. The proportion of Escherichia-Shigella, one of the genera Proteobacteria, increases with increasing disease activity. The differences in composition became more pronounced with increasing disease activity. F/B ratio in patients with ulcerative colitis as in Santoru M.L. et al. [32] decreased and, with the increase of inflammation activity, the number of Firmicutes and Bacteroidetes decreased; which correlates with inflammation and depletion of intestinal barrier function and are important therapeutic targets for the correction of dysbiosis in patients with UC [33]. Previous studies of beta diversity have demonstrated differences between healthy individuals and patients with UC [27,34], which we also found in our investigation. Our data showed significant differences between subgroups, with varying degrees of disease severity, indicating a possible connection between changes in the balance of gut bacteria and the onset and progression of UC.

Changes in the composition of the microbiome affect its production of metabolites, which has been shown in many studies [35,36,37,38]. In our study, we found that the concentration of TMAO metabolite is lower in patients with UC than in healthy subjects, which is consistent with the results of Wilson et al. [15]. As the disease activity increased according to clinical and endoscopic parameters, TMAO decreased in patients with UC. TMAO also decreased with the increase of fecal calprotectin, a marker of intestinal inflammation; and low albumin levels, which speaks in favor of the relationship between TMAO and the severity of the pathological process.

TMAO is the product of the oxidation reaction of trimethylamine (TMA) in the liver under the action of the enzyme flavin-containing monooxygenase [39]. In turn, TMA is formed from dietary choline, carnitine, and phosphatidylcholine by the action of the gut microbiota [40,41,42]. The presence of CutC/CutD genes in Firmicutes, Actinobacteria and Proteobacteria [43], CntAB in Gamma- and Betaproteobacteria and some Firmicutes (*Acinetobacter baumannii* and *Escherichia coli*) [44] and YeaW and YeaX-in taxa from Gamma- and Betaproteobacteria and several Firmicutes [45] contribute to the formation of TMA from food starting products [40,44,46]. Previously, Wilson et al. showed that while TMAO levels differed between healthy individuals and UC patients, there were no differences in choline and carnitine levels, as well as the influence of mutations in the FMO-3 gene [15]. Therefore, they suggested that TMAO levels are influenced by changes in the composition of the intestinal microbiome.

To present the knowledge gained from this study, we have investigated the mechanism of TMAO formation through changes in the gut microbiome in patients with ulcerative colitis (UC) in Kazakhstan for the first time. The accumulation of knowledge about the species and genera of bacteria that contain genes encoding enzymes promoting the formation of TMA and TMAO enables us to partially answer why TMAO levels may be altered in patients with UC. Our study has shown what changes occur in the gut microbiome of patients with UC in Kazakhstan.

We found a statistically significant decrease in the abundance of the main Bacteroides and Parabacteroides species from the *Bacteroidaceae* family, as well as an increase in the abundance of Actinomyces, Klebsiella, *Limosilactobacillus*, Streptococcus, and Escherichia-Shigella genera. In addition, we observed a decrease in representatives of the p_Actinobacteria phylum, specifically *Collinsella* and *Eubacterium* species (*Eubacterium xylanophilum* group), in patients with ulcerative colitis. The intensification of the pathological process was accompanied by a further decrease in these taxa’s abundance. Based on these findings, it can be hypothesized that the reduction in TMAO levels in patients with UC within the studied cohort may be associated with the decrease in these bacterial taxa.

We acknowledge the possibility that changes in the population of certain taxa may influence the level of TMAO in patients with UC. This suggests that alterations in the microbiome of these patients may lead to a decrease in the synthesis of precursors for TMAO production. Further research is needed to identify other microorganisms involved in the production of TMAO, and to better understand the underlying mechanisms of this process.

### 4.1. Clinical Implications

In our study, we showed how TMAO levels change in patients with UC. Given the limited specific markers of UC, TMAO can be considered a minimally invasive biomarker of the disease and its severity. At the same time, detected changes in the composition of the intestinal microbiome in UC can be the basis for the development of corrective therapy; in particular pre- and probiotics or transplantation of fecal microbiota. This would increase the efficacy of drug therapy and create a more personalized approach to the UC patient.

### 4.2. Limitations

The study was carried out on a sample of respondents living in the Central region of Kazakhstan. It should be noted that this is the first study on the composition of the intestinal microbiome and its metabolites in Kazakhstan. Further studies will expand the understanding of the microbiome of the whole country.

## 5. Conclusions

We have identified significant differences in the microbial landscape of patients with ulcerative colitis (UC), which also differ statistically significantly, depending on disease activity. We found that trimethylamine N-oxide (TMAO) levels were statistically significantly decreased in patients with UC and decreased further with the intensification of inflammation. Furthermore, we found that the microbiome of these patients underwent changes that may contribute to the decrease in TMAO production, particularly the depletion of microorganisms that produce TMAO. The results of our study provide a basis for further research into the intestinal microbiome and its metabolites in UC, as well as the potential use of TMAO as a marker of disease activity and severity.

## Figures and Tables

**Table 1:** Baseline characteristics of participants.

Variables	Ulcerative Colitis (UC)	Control (CN)	*p*-Level
Male/female, N	31 (16/5)	15 (5/10)	0.238
Age, me (Q25;Q75)	38 (33;52)	42 (33;51)	0.907
Occupation (urb/vil), N	20/11	9/6	0.767
Feeding Characteristics (in mL or gram per 1 week)			
Meat/meat products	1183	1232	0.356
Fatty fish (1 serving of 100 g)	136	145.6	0.765
Dairy products	611	653	0.493
Eggs (1 egg = 64 g.)	364	384	0.612
Cereals/Bread and bakery products	504	546.2	0.32
Fruit	331.3	688.3	<0.001
Vegetables	434	539	0.031
Alcohol (1 portion = 250 mL of beer, 100 mL of wine or 30 mL of spirits)	6 portions	1 portion	<0.001
Laboratory characteristics			
	Me (Q25;Q75)		*p*-level
Hb, g/L	131 (114;144)	134 (116;143)	0.844
ESR, mm/h	15 (4;28)	11 (8;20)	0.163
Albumin, g/L	40.8 (36.1;46.7)	44.6 (43.6;45.9)	0.016
CRP, g/L	2.3 (1;8.7)	1.1 (0.4;2.8)	0.054
Fecal calprotectin, mcg/kg	305.9 (91.1;1202)	18 (12;23)	<0.001
Clinical characteristics			
Extent, N/%:		-	-
E1 (proctitis)	3/9.7		
E2 (left-sided colitis)	13/41.9		
E3 (pan-colitis)	15/48.4		
Mayo activity, N/%:		-	-
Mayo ≤ 5 points (UC1)	8/25.8		
Mayo > 6 points (UC2)	23/74.2		

**Table 2:** Correlation of TMAO with clinical and laboratory characteristics.

	r (Spearman)	*p*-Level		r (Spearman)	*p*-Level
Age	0.29	0.113	ESR	−0.210	0.258
Defecation frequency	−0.208	0.269	CRP	−0.114	0.542
Extension	−0.018	0.924	Albumin	0.451	0.011
Hb	0.111	0.554	Fecal calprotectin	−0.458	0.010
RBC	0.198	0.285	Mayo UC-1, UC-2	−0.387	0.031

## Data Availability

Deidentified information data presented in this manuscript will be made available 6 months after publication on reasonable request by email to the corresponding author for research purposes.

## Supplementary Materials

The following supporting information can be downloaded at: https://www.mdpi.com/article/10.3390/jcm13195794/s1, Checklist of items in report.
