# Supplementary material for: Alterations of the Gut Microbiome and TMAO Levels in Patients with Ulcerative Colitis
Source: J Clin Med. 2024 Sep 28;13(19):5794. doi: 10.3390/jcm13195794 (PMC11477140; doi:10.3390/jcm13195794)
Supplement: Supplementary file 1 [file jcm-13-05794-s001.zip › jcm-3176577-supplementary.pdf]

Checklist of items in report.

	Item No	Recommendation	Page No
**Title and abstract**	1	"Alterations of the gut microbiome and TMAO levels in patients with ulcerative colitis": observational, cross-sectional study	1
		As part of a grant project AP14871959 from September 2022 to October 2023 31 patients with UC and 15 healthy volunteers over 18 years at the Clinic of NCJSC "KMU" were measured blood TMAO level and metagenomic sequencing of fecal. Results: a significant depletion of the main represent-atives of Bacteroides, Parabacteroides, Prevotella and an increase in the relative abundance of the genera Actinomyces, Klebsiella, Limosilactobacillus, Streptococcus, Escherich-iaEscherichia-Shigella were detected in patients with UC. The number of p_Actinobacteria (g_Collinsella) and p_Eubacterium (g_Xylanophilum) representatives with genes encoding TMA-trimethylamine conversion is significantly reduced in UC patients. TMAO levels were significantly lower in UC pa-tients than in healthy individuals (0.233 µmol/l, p=0.004). TMAO decreased with disease severity and was significantly differentdiffered between patients with different activities (p=0.034). Conclusions: the composition of the intestinal microbiome changes and the level of TMAO decreases in patients with UC at different activities.	
**Introduction**			
Background/rationale	2	Increasing interest in the role of the intestinal microbiome in the development and progression of ulcerative colitis has led to a number of detectable changes in most patients. A large number of studies demonstrate general and individual trends in changes in the composition of the intestinal microbiome in different countries. Also, there are prerequisites for studying the role of trimethylamine-N-oxide metabolite in UC. Summarizing the previously obtained results, we decided to study how the composition of the intestinal microbiome and the level of TMAO change in patients with UC in Central Kazakhstan at different degrees of disease activity	1-2
Objectives	3	To investigate the microbiota status and trimethylamine-N-oxide (TMAO) metabolite levels in patients with UC according to clinical and endoscopic activity	
**Methods**			
Study design	4	Observational, cross-sectional study	2
Setting	5	The studн conducted in Kazakhstan, Karaganda region in the University Clinic of the Non-commercial joint stock company "Karaganda Medical University"	2
Participants	6	The study included 46 individuals over the age of 18 with no mental or severe neurological conditions, of both genders and without restrictions based on race or ethnicity. All participants signed an informed consent form confirming their willingness to participate in the study and were screened for eligibility.	2

Variables	7	Additional criteria for inclusion in the main group included the absence of acute conditions or exacerbations of chronic diseases, as well as the avoidance of taking antibacterial medications for 6 months before the study, and non-steroid anti-inflammatory drugs (NSAIDs) and probiotics for 3 months prior. Diagnosis of ulcerative colitis included a combination clinical, laboratory, endoscopic and morphological changes	2
Data sources/ measurement	8*	Peripheral blood parameters are determined on high-performance automatic 6-diff hematological analyzers of the Sysmex XN-2000 and Beckman Coulter brands. The ELISA method is used to measure fecal calprotectin concentrations using the EliA Calprotectin 2 test system from Phadia GmBH. TMAO measured performed by High-performance liquid chromatography. A culture-free method 16s rRNA sequencing identifies and compares bacterial diversity in fecal.	2-3
Bias	9	The study was free from bias as patient recruitment was done using inclusion and exclusion criteria only. Those who performed the patient examinations and blood and fecal examinations did not have access to the participants' personal data.	
Study size	10	Based on the overall morbidity in the region, a minimum volume of participants was determined	
Quantitative variables	11	The Chi-square test was used to analyze qualitative data	
Statistical methods	12	Data entry and primary statistical processing were performed in MS Excel program of Microsoft Office software. Further, the patient's data were analyzed in IBM SPSS Statistics 22. The Kolmogorov–Smirnov (K-S test) test was used to assess the normal distribution of the data. Data with normal distribution were described by Mean with standard deviation, not normally distributed by Median and interquartile range. The Chi-square test was used to analyze qualitative data, and the Mann-Whitney U-test and Kruskal-Wallis test were used to analyze quantitative data. The Spearman correlation analysis test was utilized to calculate the mutual relationships. A p-value less than 0.05 is considered to be statistically significant (significance level α=0.05). The distribution of key study population characteristics by groups was analyzed using the Kruskal-Wallis test from the scipy libraryScipy Library version 1.13.0 and maaslin2*. Threshold values for q-value and p-value were set at < 0.05. For post-hoc analysis, Dunn's test (scikit-posthocs version 0.9.0) was used. Alpha diversity was assessed using the Shannon, Simpson, and Observed indices with the scikit-bio package version 0.6.0. Statistical data were calculated using the stats-models library version 0.14.1. Beta diversity was assessed using the Bray-Curtis and Canberra metrics, and data were transformed using the Hellinger transformation. Ordination was visualized using principal coordinate analysis (PCoA). For analysis and visualization, the scikit-bio library version 0.6.0 and matplotlib version 3.8.4 were used. PERMANOVA and ANOSIM results showed differences between groups.	4

Results

Participants	13*	46 individuals (31 UC and 15 Healthy) met inclusion and exclusion criteria at all stages of the study without dropping out. (Graphical abstract included)	Graphical abstract
Descriptive data	14*	The participants were residents living in central Kazakhstan, specifically in the Karaganda region, over the age of 18 with no mental or severe neurological conditions, of both genders and without restrictions based on race or ethnicity.	2
Outcome data	15*	46 participants of the study not dropping out	
Main results	16	A significant depletion of the main representatives of Bacteroides, Parabacteroides, Prevotella and an increase in the relative abundance of the genera Actinomyces, Klebsiella, Limosilactobacillus, Streptococcus, Escherich-iaEscherichia-Shigella were detected in patients with UC. The number of p_Actinobacteria (g_Collinsella) and p_Eubacterium (g_Xylanophilum) representatives with genes encoding TMA-trimethylamine conversion is significantly reduced in UC patients. TMAO levels were significantly lower in UC patients than in healthy individuals (0.233 μmol/l, p=0.004). TMAO de-creased with disease severity and was significantly differentdiffered between patients with different activities (p=0.034).	4-10
Other analyses	17	TMAO also decreased with the increase of fecal calprotectin, a marker of intestinal inflammation, and low albumin levels	10
**Discussion**			
Key results	18	Thus, we have identified significant differences in the microbial landscape of patients with ulcerative colitis (UC), which also differ statistically significantly depending on disease activity. We found that trimethylamine N-oxide (TMAO) levels were statistically significantly decreased in patients with UC and decreased further with the intensification of inflammation. Furthermore, we found that the microbiome of these patients underwent changes that may contribute to the decrease in TMAO production, particularly the depletion of microorganisms that produce TMAO. The results of our study provide a basis for further research into the intestinal microbiome and its metabolites in UC, as well as the potential use of TMAO as a marker of disease activity and severity	12-13
Limitations	19	The study was carried out on a sample of respondents living in the Central region of Kazakhstan. It should be noted that this is the first study on the composition of the intestinal microbiome and its metabolites in Kazakhstan. Further studies will expand the understanding of the microbiome of the whole country.	13
Interpretation	20	In our study, we showed how TMAO levels change in patients with UC. Given the limited specific markers of UC, TMAO can be considered a minimally invasive biomarker of the disease and its severity.	12
Generalisability	21	Detected changes in the composition of the intestinal microbiome in UC can be the basis for the development of corrective therapy, in particular pre- and probiotics or transplantation of fecal microbiota.	12

		This would increase the efficacy of drug therapy and create a more personalized approach to the UC patient.	
**Other information**			
Funding	22	This work was funded by the Science Committee of the Ministry of Education and Science of the Republic of Kazakhstan. Grant No. AP14871959.	
